# Functional Abdominal Pain Disorders in Children May Be Associated with Food Intolerance/Malabsorption

**DOI:** 10.3390/children10091444

**Published:** 2023-08-24

**Authors:** Wolfgang J. Schnedl, Michael Schenk, Simon Michaelis, Dietmar Enko, Harald Mangge

**Affiliations:** 1 Department of Internal Medicine, Medical University of Graz, Auenbruggerplatz 15, A-8036 Graz, Austria; 2 General Internal Medicine Practice, Dr. Theodor Körnerstrasse 19b, A-8600 Bruck, Austria; 3 Das Kinderwunsch Institut Schenk GmbH, Am Sendergrund 11, A-8143 Dobl, Austria; michael.schenk@kinderwunsch-institut.at; 4 Institute of Clinical Chemistry and Laboratory Medicine, Hospital Hochsteiermark, Vordernberger Straße 42, A-8700 Leoben, Austria; simon.michaelis@kages.at (S.M.); enko.dietmar@gmx.at (D.E.); 5 Clinical Institute of Medical and Chemical Laboratory Diagnosis, Medical University of Graz, Auenbruggerplatz 30, A-8036 Graz, Austria; harald.mangge@klinikum-graz.at

**Keywords:** functional abdominal pain disorders, irritable bowel syndrome, fructose malabsorption, histamine intolerance, lactose intolerance

## Abstract

Functional abdominal pain disorders (FAPDs) are among the most common types of chronic pain disorders in children. FAPD symptoms are characterized by chronic abdominal pain and changed bowel movements. The pathophysiology of FAPDs in children is unknown, but these conditions may have an imprecise clinical overlap to food intolerance/malabsorption. We report on 51 consecutive children (23/28 males/females; median age 15.3 years) with investigated FAPDs from 2017 to 2022 in this retrospective pilot study. Small intestinal biopsies in children demonstrated the association of lactase and diamine oxidase (DAO), which prompted us to perform hydrogen (H_2_) breath tests for lactose intolerance (LIT) and determine serum DAO for the evaluation of histamine intolerance (HIT) in pediatric patients with FAPDs. To complete the food intolerance/malabsorption evaluation tests, we included a search for antibodies against tissue transglutaminase to find celiac disease (CD), performed H_2_ breath tests to detect fructose malabsorption (FM), and conducted a search for IgA antibodies against *H. pylori* infection. The results demonstrate that all 51 children evaluated were diagnosed with food intolerance/malabsorption and/or various combinations thereof. Seven children showed FM, eight of the children had HIT, and eight children had LIT. The other children had combinations: thirteen children (25.5%) had HIT and LIT, seven children (9.8%) had FM with HIT, five children (13.7%) had FM and LIT, and three children (5.9%) had a triple combination of FM, HIT, and LIT. By describing this method of personalized investigation for food intolerance/malabsorption in children with FAPDs, we demonstrate that functional abdominal pain disorders may be associated with food intolerance/malabsorption. After such diagnosis in this pediatric population, a registered dietitian helped to establish a reduction and/or exclusion diet individually tailored to their symptomatology.

## 1. Introduction

In adults, symptoms of disorders of the irritable bowel syndrome (IBS) spectrum and pediatric functional abdominal pain disorders (FAPDs) are divided into a number of clinically distinct entities based on the Rome IV criteria. These include functional dyspepsia, irritable bowel syndrome (IBS), abdominal migraine, and functional abdominal pain that is not otherwise specified [1]. A diagnosis according to the Rome IV criteria requires the absence of any organic disease to explain the symptoms. The pathophysiology of FAPDs in children and in disorders of the IBS spectrum is unknown; therefore, current therapeutic and especially dietary measures only have unsatisfactory success [2]. In general, the available treatment options for FAPDs are limited and involve education, reassurance, lifestyle interventions, psychosocial treatment (e.g., hypnotherapy), and few experimental pharmacological compounds. To date, treatment success in pediatric patients with FAPDs is slim, and many children continue to experience their symptoms into adulthood. However, FAPDs are presumably multifactorial, and evidently have several parallels to the symptoms of the disorders of the IBS spectrum in adults [3].

Small intestinal biopsies in children demonstrated the association of lactase and diamine oxidase (DAO), which are enzymes involved in the mucosa of the small intestine in lactose intolerance (LIT) and histamine intolerance (HIT) [4]. However, functional, nonspecific, and non-allergic abdominal symptoms of the disorders of the IBS spectrum in adults have been shown to be related to food intolerances/malabsorption and *Helicobacter* (*H.*) *pylori* infection [5]. This encouraged us to use hydrogen (H_2_) breath tests for the evaluation of LIT and the determination of DAO in serum for the evaluation of HIT in pediatric patients with FAPDs. Additionally, we included an H_2_ breath test for the determination of fructose malabsorption (FM), the determination of tissue transglutaminase IgA antibodies to exclude celiac disease, and a search for IgA antibodies against *H. pylori*.

The determination of the presence of FAPDs requires a thorough individual diagnostic workup in any child, as it is the case with a personalized management approach in the symptoms of irritable bowel syndrome spectrum in adults [6]. If food intolerance/malabsorption is subsequently identified, treatment with individualized dietary plans is required. Here, we demonstrate that in children, FAPDs may be associated with single food intolerance/malabsorption or combinations thereof.

## 2. Methods

In this pilot study with retrospective evaluation in our outpatient clinic, we included 51 consecutive children with FAPDs. With a symptom duration of at least 3 months, all children reported abdominal pain, bloating, diarrhea, and soft stools. Single children reported additional heartburn, postprandial fullness, belching, the loss of appetite, and nausea, and one child had occasional emesis. The children and their parents were interviewed in detail about their eating habits and the temporal relationship of eating to the child’s symptoms. Children who completed all the tests described between the years 2017 and 2022 were included. The children had no co-morbidities and were not taking any medication.

In the morning, at the first presentation, blood drawings were performed after overnight fasting (>12 h), and the H_2_ lactose breath test was started without having chewed gum or without having performed exercise overnight. An H_2_ breath test was used to look for LIT and for FM (Gastrolyzer, Bedfont Scientific Inc., Kent, UK). H_2_ tests were performed with 200 mL of water containing 1 g of lactose per kilogram of body weight up to 50 g of lactose. End-expiratory exhalation of H_2_ was measured every 30 min (min) over a period of 150 min. Fasting blood glucose levels were determined and measured every 60 min over a 150 min period. If the H_2_ level increased by more than 20 parts per million (ppm) from baseline and/or if blood glucose increased by less than 20 mg/dL, an LIT was diagnosed. On a second occasion, the H_2_ breath test with a drink containing 0.5 g of fructose per kilogram of body weight up to 25 g of fructose dissolved in 200 mL of water was used to determine FM. If the H_2_ level increased by more than 20 ppm from baseline, a fructose malabsorption was diagnosed. The blood samples taken at the first presentation were kept refrigerated, and within 2 days, serum DAO was conducted through radio extraction assay using DAO Rea 100 (Sciotec Diagnostic Technologies, Tulln, Austria). Antibodies against tissue transglutaminase were determined with anti-tTG IgA ELISA (Euro Diagnostica AB, Malmö, Sweden). An enzyme-linked IgA immunosorbent assay anti-tTG IgA ELISA (Serion, Würzburg, Germany) was used to detect *H. pylori*.

## 3. Statistical Analysis

The statistical analyses were performed using SPSS 26.0 statistical software (SPSS Inc., Chicago, IL, USA).

## 4. Results

During this retrospective evaluation of 51 children, with a 23/28 male/female ratio, a median age of 15.3 years and an age range 6.4–18.2 years were found. The diagnosed food intolerance/malabsorption syndromes and their various combinations are presented in Table 1. A total of 23 children (45.1%) had a single food intolerance/malabsorption and 28 children (54.9%) had a combination of food intolerance/malabsorption. An FM was found in 22/51 (43.1%) of children, LIT was found in 31/51 of children (60.8%) and an HIT was found in 29/51 of children (56.9%). Neither tissue transglutaminase antibodies nor antibodies suggestive of *H. pylori* infection were detected in any of the included children.

## 5. Discussion

FAPDs are very common, with an estimated prevalence ranging up to 40% in childhood. They have a significant effect on children and their families, and are a considerable financial and socioeconomic burden. Children with FAPDs have a significantly reduced quality of life, show lower school attendance, and are one of the main reasons for pediatric consultations. To date, there is limited scientific evidence that nutritional interventions may improve the symptoms of FAPDs in children [2]. Here, we report on the results of a pilot study with the search for food intolerance/malabsorption in children with FAPDs, which subsequently allowed for the development of an approach of a personalized dietary management in these pediatric patients with FAPDs.

Childhood FAPDs appear to bear some resemblance to adult IBS spectrum disorders [1]. The diagnosis of FAPDs in children and complaints in disorders of the IBS spectrum in adults are based on the individual symptoms only because a pathophysiology is unknown. In general, the symptoms of FAPDs are also highly dependent on the subjective interpretation of the individual child. Indeed, symptoms alone are rarely, if ever, diagnostic. In adults, the symptoms of disorders within the IBS spectrum are now associated with disorders such as IBS diarrhea, IBS constipation, functional diarrhea, functional constipation, chronic functional abdominal pain, or bloating [3]. The insufficient knowledge of the pathophysiology contributes to a lack of evidence-based effective management, including unsatisfactory dietary treatments in FAPDs and in disorders of the IBS spectrum [1,2]. Nonetheless, these symptom-oriented conditions have an imprecise clinical association to food intolerance/malabsorption. In adults, ingested food components including fructose, gluten, histamine, lactose, and potentially, infection with *H. pylori* may cause abdominal complaints similar to the disorders of the IBS spectrum [5].

Lactose intolerance, appearing in 70% of worldwide populations, is mainly a genetic deficiency of the lactose-degrading enzyme lactase. In humans, lactase activity decreases after weaning, when lactose-containing breast milk is no longer part of the daily diet. Due to this downregulation of lactase, LIT may occur in children as young as five years of age. In some humans, particularly in northern Europe, lactase activity persists into adult life. However, in adults, LIT is recognized as a main cause of complaints in disorders of the IBS spectrum [7]. Approximately half of LIT patients’ GI symptoms with disorders of the IBS spectrum not only require a reduced lactose intake, but should also consider, not clearly defined, additional dietary measures to improve their symptoms [8]. However, in adults, this has been shown to be related to the presence of extra food intolerance/malabsorption [4]. Here, we demonstrate that LIT alone occurs in 16% of children with FAPDs, but various combinations of food intolerance/malabsorption with LIT were found in 61% of investigated children, as shown in Table 1.

Recently, public and scientific interest in HIT increased, and it is considered a non-allergic, adverse reaction to food. The clinical diagnosis of HIT is challenging, and there are no standardized tests for diagnosis [9]. It seems to be a consequence of mainly histamine-containing food and a supposed deficiency of the gastrointestinal (GI) enzyme DAO. In our outpatient setting, serum DAO is regularly determined in combination with the thorough anamnesis of food-related symptoms using a standardized questionnaire for the diagnosis of HIT [10]. The known genetic polymorphisms in the genes coding for DAO and histamine receptors may reason the complex various appearance of HIT symptoms in children as well as in adults. So far, HIT per se and as a GI disorder has been studied insufficiently. In the apical part of intestinal villus cells, the enzyme DAO was determined, and it is continuously released from the intestinal mucosa. DAO catalyzes the deamination of biogenic amines including histamine. Serum DAO enzyme levels have been termed as biomarkers of intestinal permeability. Small intestinal mucosal damage may decrease the DAO activity and thereby increase histamine levels [11]. Generally, more information is required to define, diagnose, and manage HIT.

Nonetheless, HIT was outlined as an extremely relevant topic in clinical pediatrics [12]. In adults and in children, the relationship between serum DAO and gastrointestinal DAO activity has not been proven, although evidence of a connection between these is accumulating [13]. The clinical presentation of HIT in children with FAPDs appears comparable to that of adults including GI symptoms in disorders of the IBS spectrum, although children present fewer symptoms than adults. HIT symptoms are defined predominantly as GI symptoms, and these also seem to be the most common complaints in children [12,13,14]. Nonetheless, only about 50% of children with low DAO activity report an improvement in symptoms with a histamine-reduced diet [15]. Here, in our evaluation, we identified 16% of children with HIT alone, but found 25% of children with FAPDs with the combination of HIT and LIT (Table 1). This seems to confirm the histological association of lactase with DAO activity that is demonstrated in the intestinal biopsies of children [4]. Consequently, HIT or LIT in children with FAPDs should not only be considered as a single food intolerance/malabsorption. Rather, it should be reflected that they may occur in combinations.

The daily consumption of fruit and/or production of sugar, with the use of high fructose corn syrup, leads to an increase in GI complaints caused by fructose. FM is characterized by an impairment of certain glucose transporters, namely intraluminal GLUT-5 and basolateral GLUT-2, in the small intestine epithelial cells. In FM, their ability to adequately absorb the monosaccharide fructose is impaired [16]. Nonetheless, the exact pathophysiology of FM mechanisms and of fructose-related symptoms in disorders of the IBS spectrum remain incompletely understood. More detailed information about FM can be found in various detailed reviews [17]. A genetic background seems to also play a role in FM, and consequently, it may cause FAPDs symptoms in children. However, we found FM alone in 14% of children with FAPDs and various combinations of food intolerance/malabsorption with FM in 43% of the investigated children (Table 1).

Celiac disease may cause indigestion and is characterized by an intestinal histologic mucosal reaction to ingested gluten in genetically susceptible individuals [18]. For celiac disease, the gluten-free diet is the only successful and essential life-long therapy. Biopsies and the histologic evaluation of duodenal mucosa are most accurate for the diagnosis of CD. However, guidelines for adults suggest screening with serologic tests for tissue transglutaminase antibodies to detect CD in disorders of the IBS spectrum patients [19]. CD occurs in approximately 1% of populations, and due to the low number of 51 children with FAPDs, we found no tissue transglutaminase antibodies.

There may be a potential association between *H. pylori* infection, which is classified as carcinogenic, and its effect on digestion in adults [6]. As in adults, it is the most common infection in children, and can lead to life-threatening complications in adulthood if not treated. Therefore, if *H. pylori* is detected, then eradication therapy is mandatory in pediatric patients [20] as well as in adults. However, non-invasive tests such as *H. pylori* IgA antibody serology are increasingly being used to rule out *H. pylori* infection [21]. If the overall prevalence of *H. pylori* infection in Austria is as low as it is in Switzerland [22], and because the prevalence of *H. pylori* infection seems to be low in children with GI complaints [20], this might explain why we did not find *H. pylori* IgA antibodies in this pilot study.

However, the small intestine makes digestive juices, which mix with bile and pancreatic enzymes to complete the digestion of proteins, carbohydrates, and fats. Undigested and unabsorbed foods and/or food components that are transported in the direction of the colon and enter the colon are used as bacterial substrates for GI microbiota, leading to fermentation. Subsequently, the duodenal microbiota already support the digestive function of the small intestine by fermenting undigested and unabsorbed food components [23].

In adult patients with complaints of the IBS spectrum, we reported on the diagnostic workup for food intolerances/malabsorption syndromes and the search for *H. pylori* infection [5]. Because of the H_2_ produced during fermentation, fructose and lactose breath tests have proven to be useful for diagnoses of FM and LIT. In pediatric populations, these breath tests are commonly used as they are non-invasive and low in cost. However, guidelines are being developed because standardization regarding indications for testing, test methodologies, and the interpretation of results is still lacking [24]. However, in our pilot study, we acknowledge that a selection bias cannot be excluded in our single-centered experience.

Most children and their parents are unable to attribute the GI symptoms of the child with FAPDs to a specific food or food component. However, if found, then single or various combinations of food intolerance/malabsorption may represent an etiological clarification for FAPDs in children. However, all of these methods need further scientific evaluations, especially in children. After performing the tests and the determination of the individual diagnoses of food intolerance/malabsorption, the children and their families received written information on the children’s individual diets.

## 6. Conclusions

Although we cannot prove causality, we show an association of FAPDs with food intolerance/malabsorption, including fructose malabsorption, histamine intolerance, and lactose intolerance. This may provide a pathophysiological explanation and may play an important role in children with FAPDs. A registered dietitian helped to develop a diet for the symptomatology with an eye towards dietary adequacy. This targeted dietary intervention with continuous reduction or exclusion diets in single or combined intolerance/malabsorption may contribute to sustainable relief.

## Figures and Tables

**Table 1:** Food intolerance/malabsorption and combinations thereof found in 51 children with FAPDs.

Food Intolerance/Malabsorption	Number of Children	Male/Female	Percent (%)
Number of children	51	23/28	100
LIT	8	3/5	15.7
FM	7	3/4	13.7
HIT	8	2/6	15.7
LIT + FM	7	6/1	13.7
LIT + HIT	13	5/7	25.5
FM + HIT	5	2/3	9.8
LIT + FM + HIT	3	2/1	5.9

Abbreviations: LIT, lactose intolerance; FM, fructose malabsorption; HIT, histamine intolerance.

## Data Availability

The data that support the findings of this study are available from the corresponding authors upon reasonable request.

## Abbreviations

DAO	Diamine oxidase
FAPDs	functional abdominal pain disorders
FM	fructose malabsorption
GI	gastrointestinal
*H. pylori*	*Helicobacter pylori*
HIT	histamine intolerance
H_2_	hydrogen
IBS	irritable bowel syndrome
LIT	lactose intolerance
ppm	parts per million
